# Calcium Buffering in the Heart in Health and Disease

**DOI:** 10.1161/CIRCULATIONAHA.118.039329

**Published:** 2019-05-13

**Authors:** Godfrey L. Smith, David A. Eisner

**Affiliations:** 1 Institute of Cardiovascular and Medical Sciences, College of Medical, Veterinary, and Life Sciences, University of Glasgow, UK (G.L.S.).; 2 Unit of Cardiac Physiology, Division of Cardiovascular Sciences, University of Manchester, UK (D.A.E.).

**Keywords:** arrhythmias, cardiac, buffers, calcium, heart failure

## Abstract

Changes of intracellular Ca^2+^ concentration regulate many aspects of cardiac myocyte function. About 99% of the cytoplasmic calcium in cardiac myocytes is bound to buffers, and their properties will therefore have a major influence on Ca^2+^ signaling. This article considers the fundamental properties and identities of the buffers and how to measure them. It reviews the effects of buffering on the systolic Ca^2+^ transient and how this may change physiologically, and in heart failure and both atrial and ventricular arrhythmias, as well. It is concluded that the consequences of this strong buffering may be more significant than currently appreciated, and a fuller understanding is needed for proper understanding of cardiac calcium cycling and contractility.

The importance of changes in intracellular calcium concentration in cardiac function needs little introduction (see^1,2^ for reviews). The systolic rise of ionized cytoplasmic calcium concentration ([Ca^2+^]_i_) activates contraction and regulates many sarcolemmal ion currents and, thereby, the electrophysiology of the cell; abnormal Ca^2+^ handling is implicated in the genesis of arrhythmias. Calcium is also a major factor in the control of gene expression. Work using fluorescent Ca^2+^ indicators has demonstrated how alterations of Ca^2+^ fluxes into the cytoplasm underlie the changes of contractility in health and disease. It is often overlooked, however, that only ≈1% of cytoplasmic Ca^2+^ is free, with the remainder being bound to cytoplasmic buffers.^3^ Therefore, the properties of these buffers will potentially play as large a role as Ca^2+^ fluxes do in determining the size and kinetics of changes of [Ca^2+^]_i_.

Here we review recent progress in characterizing cytoplasmic buffers and their effects on the physiology of cardiac muscle, and disease mechanisms, as well. It is important to note that we also highlight the numerous areas where more work is required.

## Properties of Intracellular Ca^2+^ Buffers

### Chemistry and Kinetics of Ca^2+^ Binding

Calcium (Ca^2+^) has several chemical features that make it a ubiquitous second messenger.^4,5^ It forms a chemically active divalent cation in aqueous solution with an ionic radius larger than the other common divalent ion (Mg^2+^) resulting in higher-affinity binding. The fact that its intracellular concentration is much lower than extracellular permits large changes of concentration because of sarcolemmal fluxes. Ca^2+^ binding alters the tertiary structure of proteins with consequences for their catalytic activity. This is a reciprocal interaction because binding also resists changes of the free concentration of an ion by acting as a buffer.

**Figure 1.**
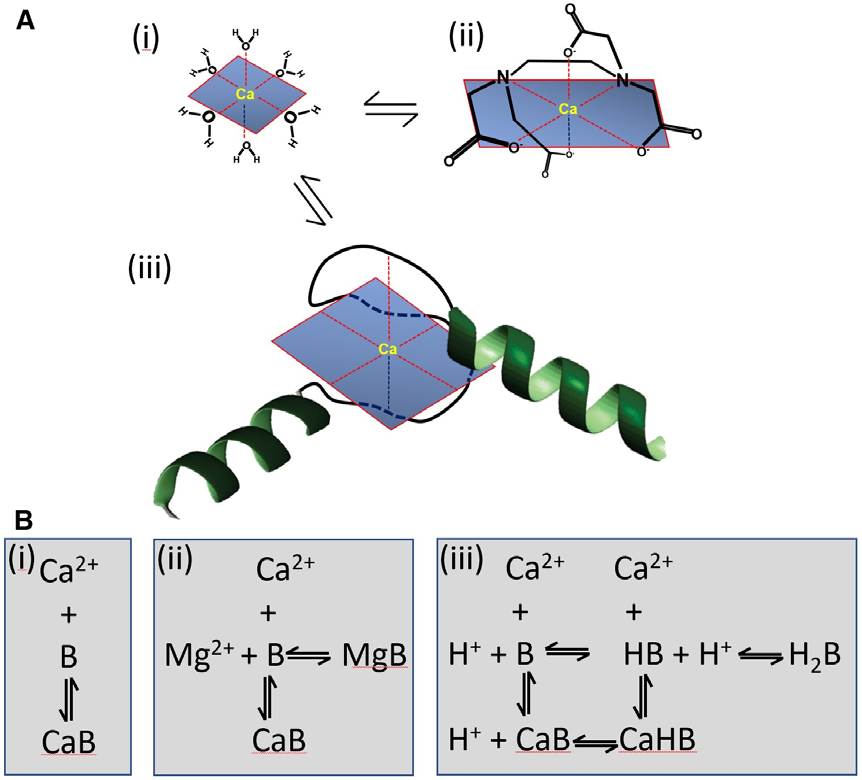
**Chemistry of Ca^2+^ binding to buffers. A**, Schematics showing Ca^2+^ in various environments: water (**i**), bound to EDTA (**ii**), and bound to an EF hand (**iii**). **B**, Reaction schemes for Ca^2+^ binding to various buffers: simple binding (**i**), competition with Mg^2+^ (**ii**), and competition with protons (**iii**).

The formation of several coordinate bonds between single Ca^2+^ ions and ligands is known as chelation. The electrons for these bonds typically come from nitrogen or oxygen atoms, replacing water molecules in the solvation sphere of the Ca^2+^ ion with a series of bonds (usually 6) in a claw-like or chelation arrangement (Figure 1A). For example, EDTA is an organic chelator designed to bind divalent cations with very high affinity. The EF hand, a helix-loop-helix configuration, is the most common Ca^2+^ binding motif in proteins.^6^ EF hand sites generally occur in pairs, and their affinity for Ca^2+^ and Mg^2+^ depends on the amino acids used to form the coordinate bonds, and the surrounding protein environment, as well. For example, calmodulin has 4 EF hand domains: (1) a high-affinity site that is normally bound at resting Ca^2+^ concentrations with a dissociation constant (*K*_*d*_) of ≈100 nmol/L, (2) 2 further binding sites on the C terminal with *K*_*d*_ values of ≈300 nmol/L.^7^ The fourth binding site has the lowest affinity (*K*_*d*_=10 µmol/L) and therefore binds negligible Ca^2+^ within the physiological range (0.1–1 µmol/L). It may have a role in controlling enzymes located near sarcoplasmic reticulum Ca^2+^ release sites in cardiac cells where [Ca^2+^]_i_ rises to ≈100 µmol/L.^7,8^

The speed of the chelation reaction depends on its complexity (Table). The fastest binding occurs with small molecules (molecular weight <1000) such as BAPTA or Ca^2+^ indicators (eg, Fura-2 or Fluo-3), with a forward rate constant that approaches the diffusion-controlled limit (minimally 10^8^ mol^-1^·L·s^-1^).^17^ Ca^2+^ binding to the EF hand structure of the regulatory site of troponin C is slightly slower.^9^ With even more complex reaction schemes (Figure 1B) that involve displacement of ions (eg, H^+^ or Mg^2+^) before Ca^2+^ binding, the kinetics slows considerably. For example, Ca^2+^ binding to the chelator EGTA requires dissociation of protons from intermediate forms of the ligand, reducing the overall forward rate constant (Table).^18^ Different forms of the EF hand motif, such as the Mg^2+^ sites of myosin, troponin C (TnC), and parvalbumin, have high relative affinities for Mg^2+^ that result in significant Mg^2+^ bound under physiological conditions.^19^ The need for Mg^2+^ to dissociate as part of the equilibration results in a low apparent rate constant of Ca^2+^ binding.

**Table.**
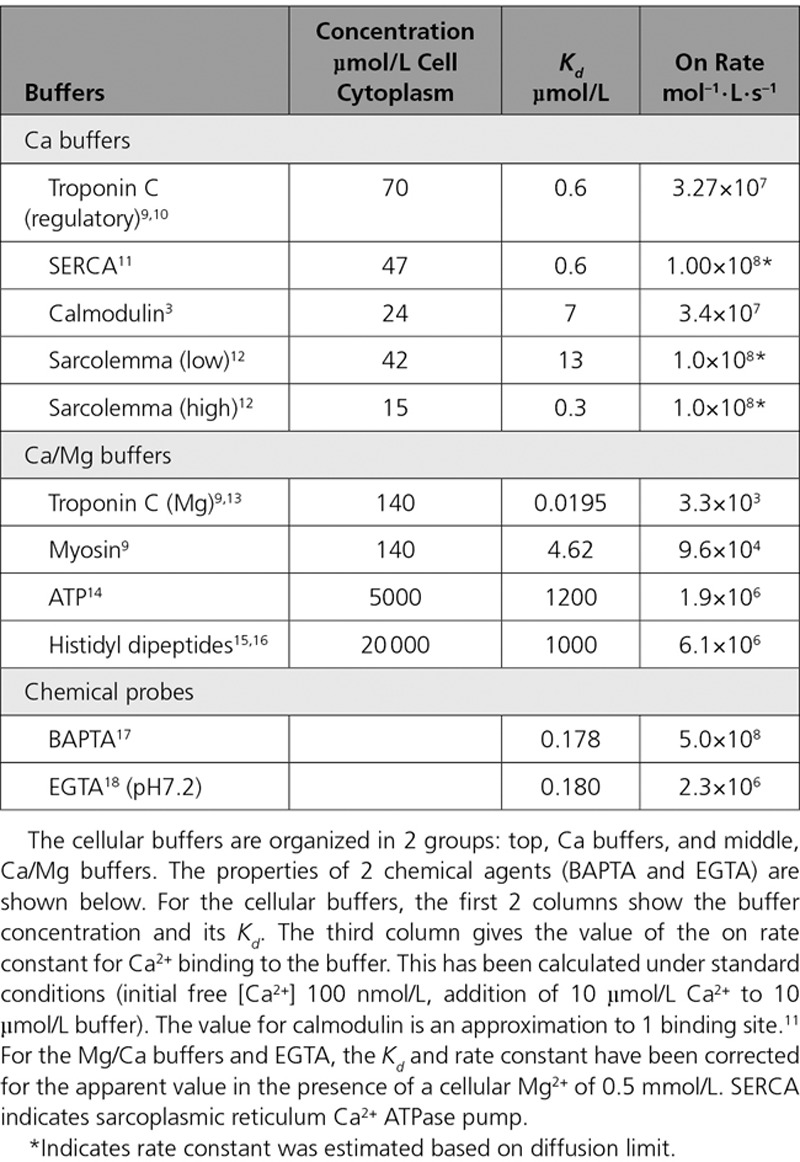
Concentration and Properties of the Major Cellular Buffers in Ventricular Myocytes

### Ca^2+^ Binding Sites in Cardiac Muscle

**Figure 2.**
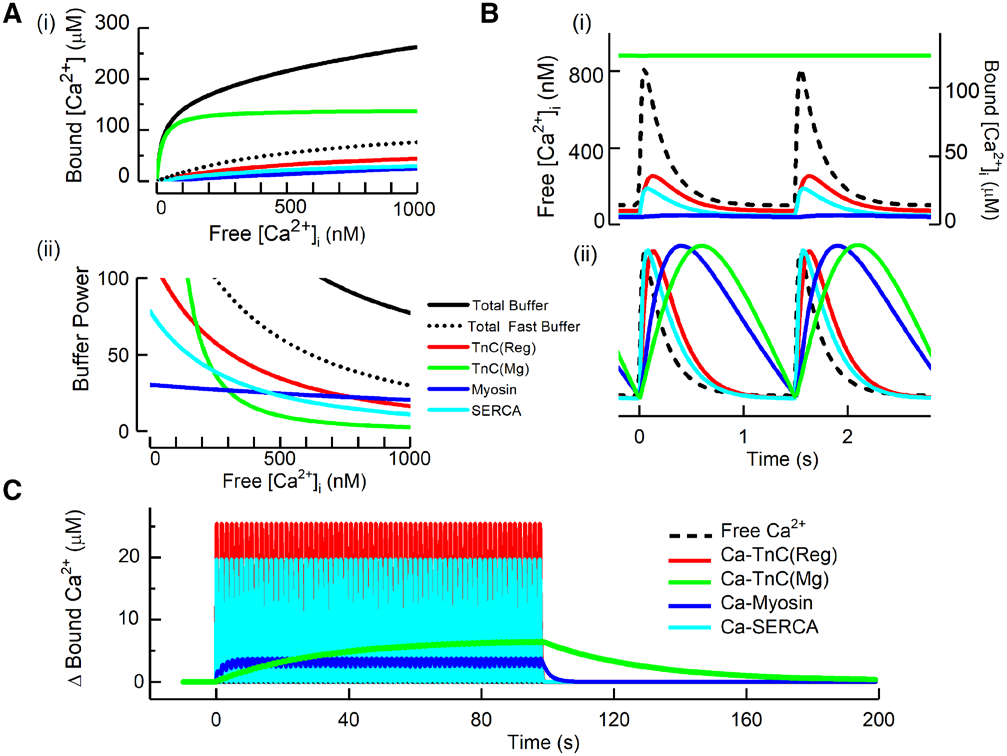
**Properties of cellular cardiac Ca^2+^ buffers. Ai**, Steady-state Ca^2+^ buffering showing the dependence of bound on free Ca^2+^. The colored curves show the contributions of the 2 Ca^2+^ binding sites (regulatory [Reg] and Mg) of TnC, myosin, and SERCA. The solid black line shows the total, calculated as the sum of these components and the others listed in the Table. The dotted black line represents the sum of the fast buffers (top part of Table). **ii**, Buffer power (calculated as in equation 2) as a function of [Ca^2+^]_i_. **B**, Time course of change of bound [Ca^2+^] in response to systolic Ca^2+^ transients applied at 1.5 Hz. **i**, Absolute levels of [Ca^2+^]. The dashed line shows free [Ca^2+^] and the colored traces show the concentration of the Ca^2+^-bound form of the various ligands. **ii**, Normalized concentrations to emphasize kinetics. **C**, The change of bound Ca^2+^ in response to a series of Ca^2+^ transients (not shown) applied at 1.5 Hz. SERCA indicates sarcoplasmic reticulum Ca^2+^ ATPase pump; and TnC, troponin C.

Based on previous work,^3,11,20^ the Table lists the major Ca^2+^ binding ligands, their estimated cytoplasmic concentrations, and dissociation constants (*K*_*d*_) alongside estimates of the rate constants of Ca^2+^ binding. The ligands that bind appreciable amounts of Mg^2+^ under physiological conditions are grouped separately. The steady-state Ca^2+^ binding for several buffers as a function of [Ca^2+^] is shown in Figure 2Ai. The 2 major contributors to buffering are TnC and sarcoplasmic reticulum Ca^2+^ ATPase pump (SERCA).

#### Troponin C

TnC has 2 classes of Ca^2+^ binding sites (Figure 2A): (1) a single, lower-affinity, regulatory site that modulates myofibril activation and thence force; and (2) 2 high-affinity sites that can also bind Mg^2+^, the Mg^2+^ sites. There is ample evidence that the affinity for Ca^2+^ of the regulatory site changes in various situations. For example, acidification decreases the binding of Ca^2+^.^21^ Work using a fluorescent TnC showed that phosphorylation of troponin I, as occurs during β-adrenergic stimulation, shifts the relationship between fluorescence and [Ca^2+^]_i_ to higher [Ca^2+^]_i_, indicating decreased Ca^2+^ affinity. A similar approach has shown that troponin and tropomyosin mutations affect Ca^2+^ affinity,^22^ and such mutations have been directly shown to affect Ca^2+^ buffering.^23,24^ It should, however, be noted that there are many circumstances (see below for discussion of heart failure) where the only available data are of a shift in the relationship between [Ca^2+^]_i_ and force. It is often not certain whether this shift results from a direct effect on Ca^2+^ binding or a subsequent step in the contraction mechanism.^25^ For example, caffeine shifts the relationship to lower [Ca^2+^]_i_ but this effect is not accompanied by increased Ca^2+^ binding.^26^ Finally, much less is known about the properties of the Mg^2+^ site on troponin than the regulatory one. One issue, which also applies to other cellular buffers, is that studies of Ca^2+^ binding are generally performed in vitro using artificial solutions as opposed to cytoplasm. Given that many cellular constituents may affect the properties of this important buffer, it is important to characterize Ca^2+^ binding to the Mg^2+^ sites under more physiological conditions. These sites can be mutated, and normal contraction requires only 1 of the 2 Mg^2+^ sites.^27^ It would be interesting to know the effects on cardiac function and Ca^2+^ cycling of the expected large decrease of Ca^2+^ buffering.

#### Sarcoplasmic Reticulum Ca^2+^ ATPase Pump

The inclusion of SERCA as a buffer emphasizes that it has 2 roles in decreasing cytoplasmic [Ca^2+^]_i_.^28^ Initial buffering by binding is followed by active sequestration. In rabbit ventricle, systole involves an increase of ≈60 µmol/L total Ca^2+^ resulting in a rise of free [Ca^2+^]_i_ of ≈0.6 µmol/L. Because of the affinity of SERCA binding sites, ≈30 µmol/L binds immediately and, with a peak uptake rate of ≈200 µmol·L^–1^·s^–1^, only ≈2 pump cycles are required to sequester the Ca^2+^ associated with a Ca^2+^ transient. This emphasizes the importance of the initial binding/buffering by SERCA in addition to its turnover in determining the rate of decay of the cytoplasmic Ca^2+^ transient.^29^

#### Other Ligands

One important distinction is whether the buffers are immobile, or can diffuse. The Table gives values for the fixed sarcolemmal binding sites. The highly diffusible ATP binds Mg^2+^ and Ca^2+^ with moderately fast kinetics, but, although present at 5 mmol/L, its low affinity results in only a modest contribution to buffering. Other diffusible ligands include creatine phosphate and histidyl dipeptides that also bind Ca^2+^ and Mg^2+^. In heart, the predominant forms of this latter group of compounds include homocarnosine and anserine with a total concentration of ≈20 mmol/L.^15^ The affinities of Ca^2+^ and Mg^2+^ for these histidyl dipeptides are lower than that of ATP, and together they constitute the bulk of the diffusible Ca^2+^ buffers. One feature of diffusible Ca^2+^ buffers is their ability to increase the apparent diffusion coefficient of Ca^2+^ through diffusion of the Ca-bound form.^30^ The histidyl dipeptides are also weak acids and contribute to the pH buffer power of the cytosol, thereby linking intracellular pH and Ca^2+^ buffering (see below).

#### Buffer Kinetics

The importance of the different kinetics of the major buffers is illustrated in Figure 2Bi. The amount of Ca^2+^ bound to the regulatory site of TnC lags slightly behind free [Ca^2+^]. The lag is much greater for the slower buffers (here the Mg^2+^ sites of myosin and TnC) and this is emphasized in the normalized data of Figure 2Bii. During a train of stimuli (Figure 2C), the slow kinetics of these buffers results in a beat-to-beat increase of bound Ca^2+^. Even at 1.5 Hz, these 2 slow sites together accumulate a total of ≈10 µmol/L Ca^2+^ and, at higher rates, when diastolic [Ca^2+^]_i_ increases, greater binding is to be expected.

## Measurement of Ca^2+^ Buffering

As discussed above, buffering depends on the summed effects of a variety of Ca^2+^ binding molecules. It is often convenient to approximate this with a composite buffer value described by a single dissociation constant and ligand concentration. The simplest method is by titration. Solaro et al^31^ studied isolated cardiac myofilaments and calculated that about 22 µmol of Ca^2+^ per kg heart is required to produce 50% maximum contraction. This was accompanied by a rise of free Ca^2+^ of ≈1.4 µmol/L, indicating that the myofilaments alone can bind >90% of the total Ca^2+^. A subsequent approach, using cardiac homogenates, found that to raise free [Ca^2+^] to 1 µmol/L required 72 µmol/kg total Ca^2+^.^32^ Hove-Madsen and Bers^33^ performed similar experiments using permeabilized cells. This removed complications of extracellular components and allowed study of mitochondrial and sarcoplasmic reticulum (SR) buffering separately from cytoplasmic. They found that cytoplasmic buffering could be described by a *K*_*d*_ of 0.42 µmol/L, plus a much lower-affinity component (*K*_*d*_=79 µmol/L).

**Figure 3.**
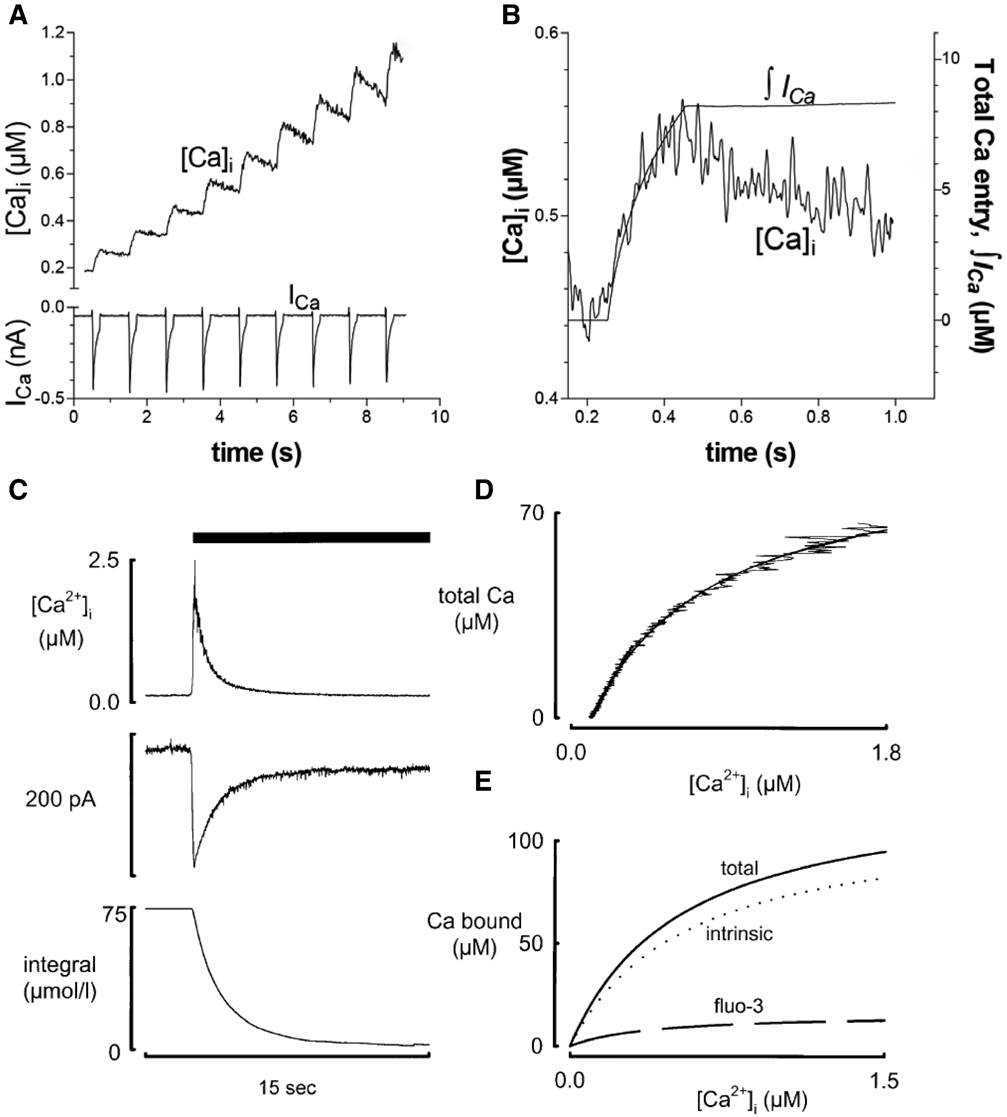
**Measurement of buffering in intact cells. A**, Comparison of the effects of Ca^2+^ influx through the L-type Ca^2+^ channel (**bottom**) with the resulting increase of [Ca^2+^]_i_. **B**, Relationship between the integral of the L-type Ca^2+^ current and the change of [Ca^2+^]_i_. Ca^2+^ removal by SR, mitochondria, NCX, and PMCA were inhibited with thapsigargin, a mitochondrial uncoupler, Na-free solution and elevated external Ca^2+^ concentration, respectively. Figure reproduced from Bers^35^ from an original article^34^ with permission. Copyright © 2001, Kluwer Academic. **C**, Determination of Ca^2+^ buffering from the caffeine-evoked release of Ca^2+^ from the SR. Traces show (from top to bottom): [Ca^2+^]_i_, NCX current, integral of current. Caffeine (10 mmol/L) was applied as shown by the bar. **D**, Relationship between total Ca^2+^ (estimated from the integral of NCX current) and [Ca^2+^]_i_. **E**, Separation of buffering into total, the contribution from the Ca-sensitive indicator (fluo-3) and the calculated intrinsic buffering of the cytoplasm. Reproduced from Trafford et al.^36^ NCX indicates sodium calcium exchange; PMCA, plasma membrane Ca^2+^ ATPase; and SR, sarcoplasmic reticulum.

The methods described above involve destruction of the cell membrane. It is also important to be able to measure buffering under physiological conditions. This was first done by depolarizing ventricular myocytes and measuring the total entry of calcium through the L-type Ca^2+^ current^20,34^ under conditions in which Ca^2+^ removal mechanisms were inhibited (see Figure 3A and 3B). Berlin et al^34^ compared Ca^2+^ entry with the rise of [Ca^2+^]_i_, giving a *K*_*d*_ of 0.96 µmol/L and a maximum buffer capacity of 123 µmol/L. A related method compared the entry of Ca^2+^ through sodium calcium exchange (NCX) with [Ca^2+^]_i_ as estimated indirectly from changes of cell length.^37^ A limitation of the method of Berlin et al is that it requires the irreversible SERCA inhibitor thapsigargin, precluding repeated measurements before and after other interventions. An alternative approach uses rapid application of caffeine to release calcium from the SR resulting in an abrupt increase of [Ca^2+^]_i_ which then decays as NCX removes Ca^2+^ from the cell. Integrating the NCX current gives a measure of the change of total Ca^2+^ concentration that is compared continuously with the change of free [Ca^2+^]_i_ to characterize buffers^36^ (Figure 3C through 3E). The caffeine response typically decays with a time constant of 1 to 2 s^38^ so this cannot detect slower buffers. In ferret ventricular myocytes, this method gave a *K*_*d*_ of 0.59 µmol/L with a maximum capacity of 114 µmol/L cell equivalent to 175 µmol/L cytoplasm^36^ and, in rat ventricular myocytes, a *K*_*d*_ of 0.49 µmol/L and a maximum capacity of 149 µmol/L cytoplasm.^39^ This is stronger Ca^2+^ buffering than that found by Berlin et al.^34^ This may be because, in part, the caffeine method includes buffering by SERCA because thapsigargin is not present. Consistent with this, addition of thapsigargin has been shown to decrease buffer power.^40^

## Buffering and the Systolic Ca^2+^ Transient

Alterations of buffering power affect the systolic Ca^2+^ transient and thence contraction. Incorporation of Ca-sensitive indicators has the side effect of increasing Ca^2+^ buffering, and this decreases systolic and increases diastolic force, and slows the rate of mechanical relaxation, as well.^41,42^ Subsequent work found a decrease of both the amplitude and rate constant of decay of the Ca^2+^ transient because, the higher the buffer power, the smaller the change of free [Ca^2+^] resulting from a given rate of Ca^2+^ pumping.^43^ Adding exogenous buffer also decreases the rate of spontaneous beating of sinoatrial node cells, presumably by decreasing the changes of [Ca^2+^]_i_ that contribute to pacemaker activity.^44^ In recent years, much work has been done using transgenic animals that express calcium indicators. In principle, the additional buffering could be a concern, but it has been demonstrated that, at the concentrations expressed, this is not an issue.^45^

The effect of increased buffering also depends on the kinetics of the added buffer. Although fast buffers simply slow the Ca^2+^ transient, slower buffers produce a biphasic decay. The initial, fast phase reflects the time taken for cytoplasmic Ca^2+^ to bind to the buffer with the slower phase depending on the kinetics of Ca^2+^ removal from the cytoplasm.^43^

It should be noted that, in the steady state, averaged over the cardiac cycle, Ca^2+^ efflux must equal influx. This efflux is determined by [Ca^2+^]_i_. If one assumes that Ca^2+^ efflux is proportional to [Ca^2+^]_i_, then, in the steady state, the decrease of amplitude of the Ca^2+^ transient resulting from increased buffering must exactly balance the slowing of decay of the transient and increased diastolic level such that the average level of [Ca^2+^]_i_ is unaffected.^46,47^ Increasing stimulation rate will load cytoplasmic Ca^2+^ buffers (Figure 2C). An interruption of beating will result in this extra Ca^2+^ being taken up by the SR and then being available for release.^23^ This may affect contractility and (see below) contribute to Ca-dependent arrhythmias. A more complicated question is what is the effect of increased buffering on SR Ca^2+^ content in the steady state? Experimental studies have found that adding exogenous cytoplasmic buffers decreases SR Ca^2+^.^43,48^ One explanation is that SERCA activity depends in a cooperative manner on [Ca^2+^]_i_,^49^ whereas NCX has a linear dependence.^50^ The decreased amplitude of the systolic Ca^2+^ transient may therefore decrease SERCA activity more than NCX, leading to a net loss of SR Ca^2+^. Further studies are required to see if the decrease of SR content with increased buffering is a general phenomenon.

## Factors that Alter Ca^2+^ Buffering

### Diastolic [Ca^2+^]_i_

**Figure 4.**
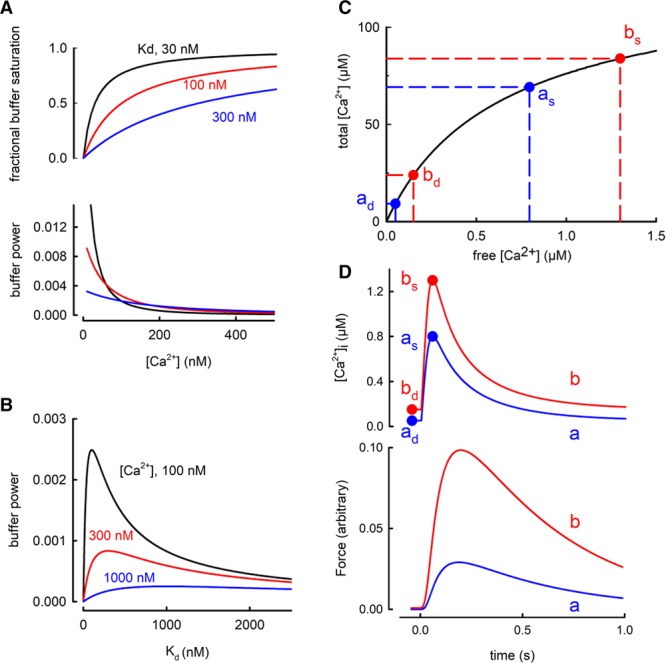
**Effects of [Ca^2+^]_i_ and buffer *K*_*d*_ on buffer power. A**, Effects of [Ca^2+^]. The top graph shows fractional saturation of a single buffer as a function of [Ca^2+^]. Curves are shown for 3 different values of *K*_*d*_. The lower graph shows calculated buffer power (change of total Ca^2+^/change of [Ca^2+^]_i_) as a function of [Ca^2+^] for these values of *K*_*d*_. Colors correspond to those above. **B**, Dependence of buffer power on *K*_*d*_ at the 3 values of [Ca^2+^] indicated. **C**, The effects of a small increase of diastolic [Ca^2+^]_i_ on the change of systolic [Ca^2+^]_i_ produced by the addition of a fixed amount of total [Ca^2+^]. The buffer curve (fast buffers only) shows 2 levels of diastolic [Ca^2+^]_i_ indicated as a_d_ (50 nmol/L) and b_d_ (150 nmol/L). The systolic levels (respectively, a_s_ and b_s_) are obtained by adding 60 µmol/L total [Ca^2+^] resulting in a larger increase of systolic [Ca^2+^]_i_ in b compared with a. **D**, Simulation of the effect of adding and removing 60 µmol/L total Ca^2+^ with kinetics designed to represent a Ca^2+^ transient. Labels correspond to points in **C**. **Upper**, simulated Ca^2+^ transients; **Lower**, predicted force responses calculated using a published model.^53^

For a simple buffer, total ([Ca_T_]) and free ([Ca^2+^]) are related by:


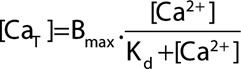
(1)

where *B*_max_ is the total buffer concentration and *K*_*d*_ is the concentration of Ca^2+^ at which 50% of the buffer has Ca^2+^ bound. The upper graph of Figure 4A shows such relationships for 3 values of *K*_*d*_.

Buffer power (β) is defined as the change of total Ca^2+^ divided by that of free Ca.


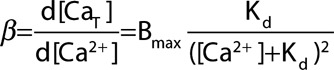
(2)

The individual contributions of the major individual buffers to the total buffer power are shown in Figure 2Aii. At [Ca^2+^]_i_ ≈100 nmol/L, the Mg^2+^ sites on TnC make the largest contribution, whereas, at >200 nmol/L, these are tending to saturation, and the regulatory site and SERCA contribute most. The lower graph of Figure 4A shows that buffer power has its highest value (equal to *B*_max_/*K*_*d*_) at low [Ca^2+^] and decreases as [Ca^2+^] increases. When [Ca^2+^]=*K*_*d*_ the buffer power is 30% of the value at 0.1 *K*_*d*_ and, at 2 *K*_*d*_, it is only 13% of this level. Consequently, the greater the diastolic level of [Ca^2+^]_i_, the larger will be the increase of [Ca^2+^]_i_ produced by a given release of total Ca from the SR.^51,52^ Some appreciation of the importance of this effect is provided by the buffer curve of Figure 4C. Because of the flattening of the buffer curve at elevated [Ca^2+^]_i_, an increase of 60 µmol/L total [Ca^2+^] produces a larger increase of free [Ca^2+^] when applied from a higher diastolic [Ca^2+^]_i_ than from a lower. This is clear in the simulated transients of the upper graph of Figure 4D. An increase of diastolic [Ca^2+^]_i_ of only 100 nmol/L (from 50 to 150) increases systolic [Ca^2+^]_i_ by 500 nmol/L. Therefore, an increase of diastolic [Ca^2+^]_i_ alone can lead to an increase of systolic, which is predicted (see lower graph Figure 4D) to result in a large increase of developed force with little change of resting force. Finally, the decrease of buffer power at elevated [Ca^2+^]_i_ has also been suggested to account for a rapid initial rate of decay of the Ca^2+^ transient.^54^

This consequence of changes of diastolic [Ca^2+^]_i_ will add to the inotropic effects of manoeuvers such as the addition of cardiac glycosides^55^ or β-adrenergic stimulation^56^ that can increase diastolic [Ca^2+^]_i_. It is also a possible explanation for changes of the amplitude of the Ca^2+^ transient and force under the many conditions where there are no measurements of diastolic [Ca^2+^]_i_. Testing this will require obtaining and comparing absolute measurements of [Ca^2+^]_i_ between cells or tissues from different animals or patients. There is a dearth of such measurements in the literature^57^ because it is much easier to measure changes of fluorescence of a Ca^2+^-sensitive indicator than absolute levels of [Ca^2+^]_i_. Properly calibrated measurements, ideally using ratiometric indicators, are required.

### Stimulation Rate

Repetitive stimulation will load slower Ca buffers (Figure 2C). This will decrease buffer power and might therefore increase the rise of [Ca^2+^]_i_ produced by a given increase of total cytoplasmic Ca^2+^, contributing to the inotropic effects of increased rate.^52^ This effect is analogous to that discussed above for elevated diastolic [Ca^2+^]_i_, but the slow kinetics result in a memory so that, following a change of rate, the effects on systolic [Ca^2+^]_i_ and thence on the action potential duration may outlast those of diastolic [Ca^2+^]_i_. Such effects may also contribute to the slow effects of changes of rate on parameters such as action potential duration.^58^

### Buffer *K*_*d*_

Equation 2 (Figure 4A) shows that, at lower values of [Ca^2+^]_i_, buffer power is greater the lower the value of *K*_*d*_ because this results in stronger Ca^2+^ binding. In contrast, at higher [Ca^2+^]_i_, the lower the *K*_*d*_, the less the buffer power as the buffers become saturated (see^23^ for experimental demonstration). Figure 4B shows the biphasic dependence of buffer power on *K*_*d*_ with the maximum being reached when *K*_*d*_=[Ca^2+^]_i_. Therefore, increasing buffer affinity will increase buffering at diastolic levels of [Ca^2+^]_i_ but decrease it at peak systolic ones.

One issue that has received no attention is whether Ca^2+^ buffering is the same in all cells in the ventricle. Given the regional differences of expression of other proteins including pumps^59^ and channels,^60^ heterogeneity of buffering would not be surprising. Likewise, possible variations of Ca^2+^ buffering between individuals, because of mutations and polymorphisms, do not appear to have been considered.

## Physiological Modulation of Buffering

### β-Adrenergic Stimulation

The 2 major Ca^2+^ buffers, TnC and SERCA, are regulated by the phosphorylation of troponin I and phospholamban, respectively, resulting in increased affinity of Ca^2+^ for SERCA^61^ and decreased affinity of Ca^2+^ for TnC.^62^ One might therefore expect that β-adrenergic stimulation would alter the buffer power. Experimental measurements, however, found no such effect,^40^ possibly because of 2 opposing factors: phosphorylation increases the affinity of Ca^2+^ binding to SERCA, but lowers it for troponin. If the *K*_*d*_ values are above the range of [Ca^2+^]_i_ considered, these effects will respectively increase and decrease buffer power (Figure 4A). Subsequent experiments, performed on transgenic mice in which either troponin could not be phosphorylated or lacking phospholamban, found the expected increase and decrease, respectively, of buffer power on phosphorylation. Further work is required to investigate the possibility that, in other species, the balance is less exact, and, therefore, phosphorylation may have a net effect on buffer power. As mentioned above, it should also be noted that the effects of a change of Ca^2+^ affinity on buffer power will depend on the range of [Ca^2+^]_i_ under investigation.

### Effects of Changes of pH on Buffering

Many Ca^2+^ buffers can bind protons as an alternative to Ca^2+^ ions. Direct measurements have shown that acidification decreases Ca^2+^ binding to troponin.^21^ Therefore, acidification will decrease the affinity for Ca^2+^ with a decrease of Ca^2+^ buffering power predicted at values of [Ca^2+^]_i_ below the *K*_*d*_ (Figure 4B). It is surprising that intracellular acidification had no effect on Ca^2+^ buffering.^63^ We suggest that this may occur because, although a decrease of Ca^2+^ affinity of low-affinity buffers will decrease buffer power, decreased affinity of very-high-affinity buffers will increase their contribution to buffering. Acidification has been shown to increase resting [Ca^2+^]_i_ in rat ventricular myocytes, an effect attributed to displacement of Ca^2+^ from buffers.^15^ It is not clear, however, why such displacement should produce the observed maintained increase of [Ca^2+^]_i_; one would expect a transient increase that decays back to baseline as Ca^2+^ is pumped out of the cell. It may result from the inhibition of Ca^2+^ efflux on NCX by acidification.^64^ If this is the case then the maintained effect on [Ca^2+^]_i_ is presumably a consequence of the NCX effect and not of altered buffering. This question could be resolved by directly measuring the effects of pH on NCX activity.

## Cardiac Dysfunction and Ca^2+^ Buffering

### Atrial Buffering, Fibrillation, and Failure

**Figure 5.**
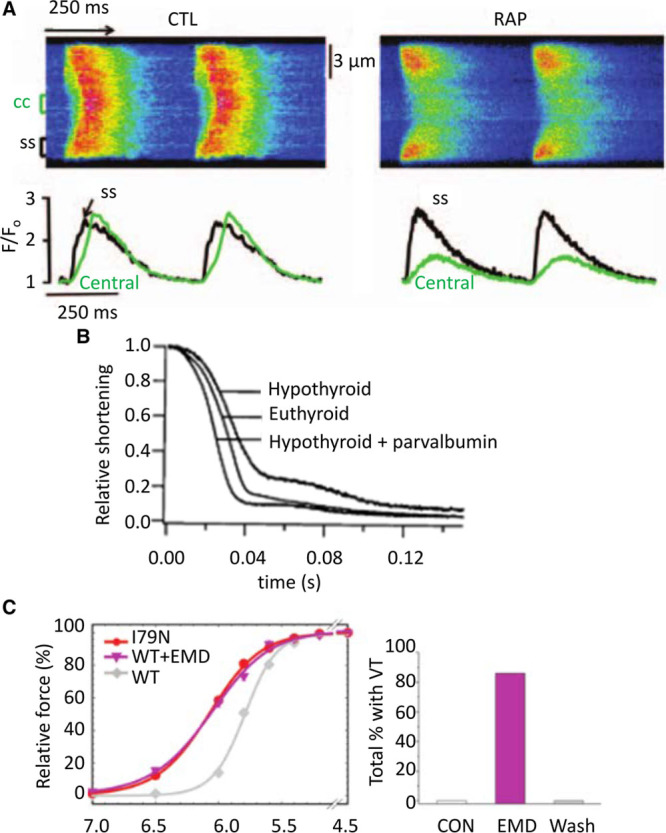
**Effects of altered buffer power. A**, Abolition of centripetal propagation of the Ca^2+^ transient by rapid atrial pacing (RAP). Upper traces are linescan images. Lower records show [Ca^2+^]_i_ measured at surface of cell (black) and in center (green). **Left**, Records from control. **Right**, Records after rapid stimulation. Reproduced from.^71^ **B**, Relaxation of contraction in isolated rat myocytes. Records show control (euthyroid), hypothyroid, and hypothyroid with parvalbumin expressed by gene transfer. Reproduced from.^72^ **C**, Left, comparison of the effects of the I79N mutation of troponin T with that of the Ca^2+^ sensitizing agent EMD 57033 on the relationship between pCa and force. **Right**, Effects of EMD 57033 on the occurrence of ventricular tachycardia (VT). Reproduced from Greiser et al.^73^

The total concentration of Ca^2+^ buffers in rat atrial myocytes has been reported to be about 3 times greater than in ventricular myocytes with no difference in apparent *K*_*d*_,^65^ possibly because of higher SERCA expression in the atrium than in the ventricle. Changes in Ca^2+^ buffering have been suggested to be important in both normal and abnormal atrial function. For example, in sheep atria, buffer power increases with age because of an increase of Ca^2+^ affinity of the buffers.^66^ This decreased both the amplitude and rate of decay of the systolic Ca^2+^ transient. Atrial myocytes from many species, including rabbits and cats, have few or no t-tubules (^67^ for review) and the systolic Ca^2+^ transient begins at the periphery of the cell and then propagates toward the center.^68,69^ Increasing Ca^2+^ buffering by incorporation of EGTA can prevent this propagation.^70^ A modeling study also predicted this inhibitory effect of high buffer concentrations but pointed out that lower concentrations of mobile buffers such as ATP facilitate propagation.^30^ Greiser et al^71^ investigated the effects of rapid atrial pacing in rabbits to mimic the effects of atrial fibrillation. This resulted in a 2- to 3-fold increase of buffering power, at least in part, because of decreased phosphorylation of troponin I which was accompanied by (Figure 5A) decreased centripetal propagation. Evidence for a causal link between increased buffering and decreased propagation was provided by showing that incorporation of BAPTA to increase buffering mimicked the effect on propagation. The effects of rapid pacing to induce heart failure have also been studied on sheep atrial myocytes where a decrease of buffer power was observed.^74^ This was accompanied by a decrease of the amplitude of the central calcium transient attributed to the loss of transverse tubules rather than a change of buffering.^75^ A similar decrease of calcium buffering in the sheep has been observed during atrial fibrillation where it was suggested to lead to arrhythmogenic Ca^2+^ waves, thereby contributing to atrial fibrillation.^76^ More work is required on changes of atrial Ca^2+^ buffering and their importance in atrial function. Finally, it is worth noting that the atrial studies reviewed above could not exclude the effects of small changes of end-diastolic [Ca^2+^]_i_ on the measured buffer power.

### Ca^2+^ Buffering and Heart Failure

Ca^2+^ buffering is unaffected by pacing-induced heart failure in both dogs^77^ and sheep.^78^ In contrast, in samples from human ventricle, the Ca^2+^ sensitivity of contraction was increased in dilated cardiomyopathy, possibly because of the decreased phosphorylation of troponin I.^79^ Increased Ca^2+^ sensitivity was also found in canine dilated cardiomyopathy^80^ and mouse infarct models.^81,82^ As mentioned earlier, changes of Ca^2+^ sensitivity of contraction do not necessarily indicate altered Ca^2+^ binding and buffering; direct measurements of Ca^2+^ binding are therefore required. Increased myofilament Ca sensitivity by itself will decrease cardiac relaxation and thereby contribute to diastolic heart failure. In addition, any consequential increase of Ca^2+^ buffering will slow the decay of [Ca^2+^]_i_, worsening relaxation. In contrast to the data discussed above, either the induction in rats of pressure overload–induced left ventricular hypertrophy or heart failure following myocardial ischemia resulted in a decreased Ca^2+^ sensitivity for activation of contraction, an effect attributed to alterations in troponin.^83^ Some of the controversies in this area have been reviewed.^84^ As far as myocardial ischemia is concerned, it is well known that troponin is lost from the heart and, indeed, the appearance of troponin I and troponin T in plasma is diagnostic of cardiac damage. Troponin release has also been detected in myocardium in conditions not associated with obvious cellular degeneration, but this only represents a small fraction (≈3%) of the total troponin^85^ and will not therefore significantly affect cellular buffering.

Finally, it should be noted that many studies of heart failure find a decrease of SERCA expression.^86^ We speculate that the consequent decrease of Ca^2+^ buffering would compensate in those situations where an increase of myofilament buffering is expected and worsen where there is a decrease. Again, it will be important to repeat these studies of heart failure while measuring buffering directly.

It is also important to reemphasize the potential effects (Figure 4C) of changes of diastolic [Ca^2+^]_i_ and, thence, of buffering power on the amplitude of the calcium transient. A major problem here is the paucity of measurements of diastolic [Ca^2+^]_i_. It is essential that studies on heart failure ask the simple question: how accurately has diastolic [Ca^2+^]_i_ been measured and can it be excluded that changes (for example, between animals or in disease) account for the observed changes of systolic [Ca^2+^]_i_?

Work from Metzger and colleagues has demonstrated that changes of Ca^2+^ buffering may not simply be involved in the development and consequences of heart failure, but may also be used to treat it. They suggested that impaired relaxation in heart failure could be ameliorated by adding intracellular buffers. They noted that fast Ca^2+^ buffers slow both the rise and fall of [Ca^2+^]_i_ and decrease the amplitude of the Ca^2+^ transient, and, instead, they advocated the use of parvalbumin, a skeletal muscle Ca^2+^ buffer. This has the important property that it binds Ca^2+^ slowly because Mg^2+^ has to dissociate first and, therefore, there is little attenuation of the peak Ca^2+^ transient. It will bind Ca^2+^ during diastole thereby improving diastolic performance. Incorporation of α-parvalbumin was shown to accelerate the decay of [Ca^2+^]_i_ with no effect on peak [Ca^2+^]_i_ and (see Figure 5B) also reversed the slowing of relaxation produced by experimental hypothyroidism^72^ and in the Dahl salt-sensitive rat model of diastolic dysfunction.^87^ Subsequent work has turned to altering the structure and thence the relative Ca^2+^ and Mg^2+^ affinities of parvalbumin analogs to improve the effects.^88,89^ In general, these effects of parvalbumin highlight the potential importance of endogenous slow buffers such as the Mg^2+^ site of TnC and myosin.

### Ca^2+^ Buffering and Hypertrophic Cardiomyopathy

Several studies have examined the molecular basis of familial hypertrophic cardiomyopathy (FHC). Much of this work involves the effects of mutations in thin filament proteins such as troponin and tropomyosin, which are among the causes of FHC. Robinson et al^22^ showed that mutations causing hypertrophic cardiomyopathy increased the binding affinity of Ca^2+^ to myofilaments (as assessed with a fluorescent troponin) and presumably therefore Ca^2+^ buffering. They proposed that alterations of buffering might lead to pathological changes of the Ca^2+^ transient. Troponin mutations were subsequently investigated in a mouse model of the related condition of restrictive cardiomyopathy and the predicted decreased amplitude and slowed decay of the Ca^2+^ transient observed.^23,90^ In addition, myofilament Ca^2+^ sensitization with EMD 57033 mimicked the effects of troponin T mutations on Ca^2+^ buffering and the Ca^2+^ transient.^23^ A recent study used adenovirus to infect isolated myocytes with troponin or tropomyosin mutations and, again, found an increase of diastolic [Ca^2+^]_i_.^24^ Although the above results would be expected from an increase of buffering power, it has been reported that there is a decrease of SERCA expression that may also contribute.^91^ This study also found that the late Na^+^ current inhibitor ranolazine abolished the slowing of decay of the Ca^2+^ current. Although no data are available, it seems unlikely that ranolazine would affect Ca^2+^ buffering. It may therefore be that some of the effects of thin filament mutations are directly attributable to Ca^2+^ buffering, and others are a secondary consequence of the resulting heart failure, possibly attributable to decreased SERCA.

As mentioned in an earlier section, the Mg^2+^ sites on troponin are important contributors to buffering at low [Ca^2+^]_i_. It is therefore interesting that one of the mutations associated with FHC (D145E) greatly decreases the affinity of Ca^2+^ binding to these sites.^92^ At first sight, this might appear to contrast with the association between FHC and the increased affinity reviewed above. These observations may be reconciled by noting that a decrease in affinity of the very-high-affinity Mg^2+^ TnC sites will actually increase Ca^2+^ buffering power in the systolic range of [Ca^2+^]_i_.

### Ca^2+^ Buffering and Arrhythmias

Ventricular arrhythmias constitute a major cause of death in FHC.^93^ The Knollmann group has investigated the underlying mechanisms in transgenic mice. Incorporation of mutations in troponin T or tropomyosin led to ventricular tachycardia. These mutations also sensitized the contractile machinery to activation by Ca^2+^ (Figure 5C) with those that produced the greatest incidence of ventricular tachycardias and arrhythmias having the greatest Ca-sensitizing effect.^73^ A causal link between Ca sensitization and arrhythmogenesis was provided by showing both that EMD 57033 caused arrhythmias and the contractile uncoupler blebbistatin decreased both Ca sensitivity of the contractile machinery and arrhythmia susceptibility. These arrhythmias were accompanied by a shortening and triangulation of the action potential, and electric repolarization alternans, as well (see below). Subsequent work using myocytes derived from human-induced pluripotent stem cells reproduced these effects of increased Ca^2+^ buffering by myofilaments on action potential shape and suggested that the shortened, triangulated action potential could be attributable to increased buffering decreasing the amplitude of the systolic Ca^2+^ transient and thereby the inward (depolarizing) NCX current.^94^ Although this is an attractive explanation, it is also worth noting that (see above), as well as decreasing the amplitude of the Ca^2+^ transient, increased buffering slows decay, making it harder to predict the net effect of increased buffering on NCX current. Another article showed that, when regular pacing was terminated by a pause, the next Ca^2+^ transient was larger than control and this effect was more prominent in troponin T mutations that sensitize to activation by Ca^2+^.^23^ This effect was attributed to a higher cell Ca^2+^ content in the mutant during stimulation, with the excess Ca^2+^ being taken up by the SR such that release after a pause results in a prolonged action potential, increasing the probability of an arrhythmogenic early afterdepolarization. Any increase of diastolic [Ca^2+^]_i_ and consequent decrease of buffer power may also increase the rise of [Ca^2+^]_i_ caused by release from the SR. In the work reviewed above, the increase of buffering was a consequence of genetic changes. Similar increases of myofilament Ca^2+^ sensitivity have been reported following myocardial infarction^81^ where manoeuvers that decrease Ca^2+^ sensitivity were found to abolish ventricular tachycardia following pauses of stimulation. As reviewed above, an increase of Ca^2+^ buffering is proarrhythmogenic. It has also been shown, however, that addition of the buffer EGTA can prevent the propagation of arrhythmogenic Ca^2+^ waves,^95^ and therefore the net effect may be more complicated.

The induction of alternans of the action potential duration by increased Ca^2+^ buffering attributable to thin filament mutations^73^ may be a consequence of the slowed decay of the Ca^2+^ transient, resulting in incomplete recovery at the time of the next stimulus at increased rates. This would be analogous to the idea that the increased propensity of endocardium in comparison with epicardium to alternans is associated with a more slowly decaying Ca^2+^ transient because of lower SERCA expression.^59^ In contrast, a modeling study has predicted that increasing cytoplasmic Ca^2+^ buffering should decrease the probability of alternans occurring by decreasing the probability that Ca^2+^ released from the SR induces further Ca^2+^ release from neighboring release sites.^96^ This is consistent with the experimental demonstration, in whole mouse hearts, that addition of the buffer EGTA decreased the occurrence of alternans^48^ and may be related to the experimental observation that increasing cytoplasmic Ca^2+^ buffering decreases the frequency of propagating Ca^2+^ waves^97^ and makes Ca^2+^ sparks terminate earlier.^98^ Further work is clearly required in understanding the relationship between Ca^2+^ buffering and alternans.

### Why Do Cells Have Ca^2+^ Buffers?

A high level of Ca^2+^ buffering is not unique to cardiac myocytes. For example, ≈99% of the Ca^2+^ entering chromaffin cells binds to cytoplasmic buffers.^99^ The presence of Ca^2+^ buffers means that much larger movements of total Ca^2+^ are required to produce a given change of [Ca^2+^]_i_. Given that calcium movements account for up to 30% of the total energy consumption of the heart,^100^ one might wonder why evolution has resulted in such strong buffering. There are several explanations. (1) It may be an inescapable consequence of the fact that using Ca^2+^ as a second messenger requires high concentrations of Ca^2^^+^ binding proteins, for example, to activate contraction. (2) A high Ca^2+^ buffering may stabilize Ca^2+^ signaling by stopping an abnormal increase of [Ca^2+^]_i_ in one part of a cell propagating throughout the cell. In this context, it is worth noting that (Figure 2Aii) the dependence of buffer power on [Ca^2+^]_i_ means that the buffer power is much lower in systole than diastole. This may help Ca^2+^ release during systole, but protect against it in diastole. (3) The need for buffering may relate to the low intracellular concentration of calcium. A diastolic concentration of 100 nmol/L equates to 6×10^16^ ions per liter corresponding to a mean distance between ions of 0.25 µm. Soeller and Cannell^8^ have modeled Ca^2+^ fluxes into the space between the transverse tubule and SR (dyad). They calculated that, at a concentration of 100 nmol/L, each dyad would contain between 0.007 and 0.028 Ca^2+^ ions. At 10 µmol/L, there will be between 0.7 and 2.8 ions. This would make it impossible to control [Ca^2+^]_i_ in a stable manner because a single Ca^2+^ transported into or out of the space would result in an enormous fractional change of [Ca^2+^]_i_. As pointed out previously by Bers,^35^ at such low concentrations, chance will determine whether a transporter interacts with an ion. In contrast, if total Ca^2+^ is 100 times the free then there will be between 70 and 280 ions per cleft. (4) If troponin was the only buffer, then virtually all the total Ca^2+^ would be bound to troponin irrespective of its *K*_*d*_. This would make it impossible to change force by altering *K*_*d*_ because this requires other buffers to take up a fraction of the total Ca.

## Conclusions

The concentration of buffered calcium in cytoplasm is 2 orders of magnitude greater than that of the free concentration, and, therefore, the buffers have an enormous effect on calcium signaling. There is a need for more work investigating whether changes of buffer properties, either directly or secondary to changes of diastolic [Ca^2+^]_i_, contribute to alterations of calcium handling and contractility. The limited human data reviewed above and extrapolated from animal models argue that changes of Ca^2+^ buffering are important in determining both inotropy and proarrhythmic status in conditions such as cardiomyopathies (dilated cardiomyopathy and hypertrophic cardiomyopathy) and ischemic heart failure in both health and disease. Clarification will require more work on human tissue.

## Acknowledgments

We are indebted to F. Burton and S. Wray for comments on an earlier version of the manuscript and to Q. Lachaud for the design of Figure 1.

## Sources of Funding

This work from is supported by grants from the British Heart Foundation (PG/17/12/32847 to Dr Smith and CH/2000004/12801 to Dr Eisner).

## Disclosures

None.
